# Geometry, shunt, and recirculation in hollow-fiber oxygenators

**DOI:** 10.1051/ject/2025066

**Published:** 2026-09-15

**Authors:** Ignazio Condello, Youssef El Dsouki

**Affiliations:** 1 School of Medicine and Surgery, University of Insubria Via Ravasi, 2 21100 Varese VA Italy; 2 Maastricht University Medical Centre (MUMC) Maastricht The Netherlands

Dear Editor

In clinical extracorporeal circulation, hollow-fiber oxygenators are usually evaluated for long-term performance on gas transfer capacity, pressure drop, and priming volume. However, the phenomena of shunt fraction and recirculation are equally relevant to the perfusionist’s daily practice. The shunt may be defined as the portion of blood that traverses the device with limited or no exposure to the functional fiber surface, while recirculation represents blood volume that loops internally within the fiber bundle, the heat exchanger, or the arterial filter before returning to forward flow. These hidden dynamics directly influence oxygen transfer, pressure readings, hemocompatibility, and embolic safety [1]. The geometry of the oxygenator is one of the key determinants of shunt and recirculation. Cylindrical configurations guide blood longitudinally or radially around the fiber bundle, promoting relatively uniform flow but also prone to creating preferential channels of low resistance. Parallelepiped or plate-type designs distribute blood in a rectangular housing, often in cross-flow fashion, increasing mixing and surface contact, though sometimes generating stagnation zones at the periphery [2]. Spiral-wound geometries**,** where fibers are packed in helical fashion, provide compact housings with low pressure drops, but can favor internal recirculation paths if the inlet distribution is not perfectly balanced. In some designs, radial or elliptical flow arrangements are employed to enhance homogeneity, yet these too may create high-shear focal regions and potential internal loops. The heat exchanger and the arterial filter also contribute, with polymeric or metallic exchangers shaping resistance and shear, and filter meshes inevitably generating vortices that may prolong bubble residence or alter embolic clearance [3]. Beyond geometry, the role of monitoring becomes crucial (Figure 1).

**Figure 1:**
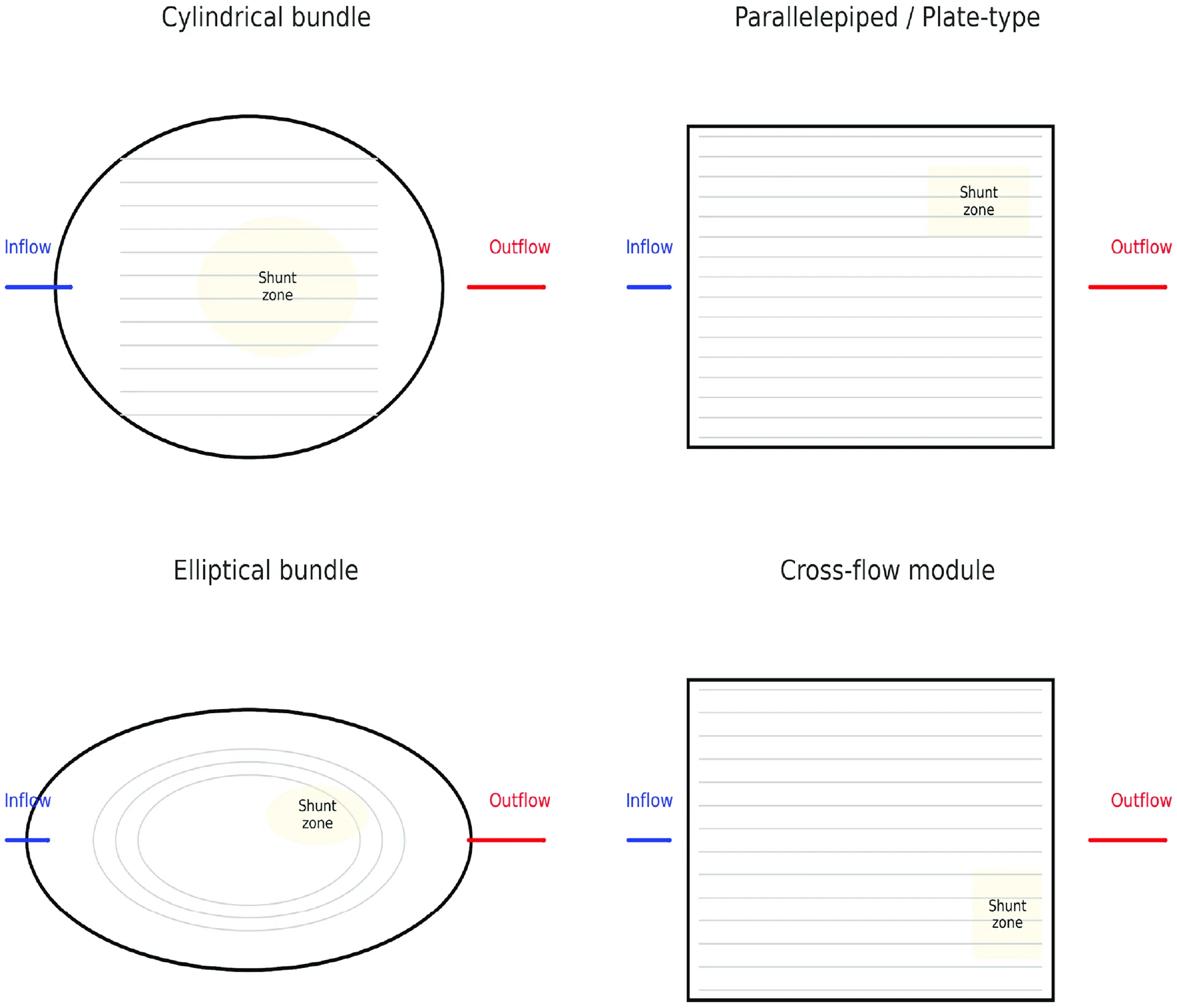
Schematic example representation of internal shunt and recirculation zones in hollow-fiber oxygenator geometries.

For the perfusionist, it is not sufficient to observe only the pressure gradient across the integrated system or the single oxygenator. The use of flowmeters placed at the inlet and outlet can help quantify flow discrepancies, providing an indirect measure of shunt or recirculation. A simple and practical way to express this is:

$$S=1-\frac{Q_{\mathrm{out}}}{Q_{\mathrm{in}}},$$

where *Q*_in_ is the blood flow entering the oxygenator, and *Q*_out_ is the flow measured at its outlet. This formula essentially expresses the fraction of blood that does not appear at the outlet relative to the inlet*.* If inlet and outlet flows are identical, *S* = 0 meaning no measurable shunt or recirculation. If the outlet flow is lower, the difference is interpreted as blood volume that either recirculates inside the device or bypasses effective membrane surfaces. Clinically, values very close to zero are considered optimal, while results above 5–10% suggest a significant degree of shunt or recirculation. In particular, analyzing the shunt fraction in the context of different pump technologies may open new perspectives. With roller pumps**,** flow delivery is pulsatile or not and relatively fixed by the mechanical action of the rollers, while with centrifugal pumps**,** flow is preload- and afterload-dependent, making shunt and recirculation phenomena more dynamic and potentially more difficult to detect without careful monitoring. This technical consideration finds an intriguing physiological parallel. In the human body, shunt fractions exist both physiologically and pathologically: small intrapulmonary shunts are part of normal physiology, while atrial or ventricular septal defects are quantified by the *Qp*/*Qs* ratio to express pathological left-to-right or right-to-left shunting. While this analogy helps to conceptualize extracorporeal shunt phenomena, it must be acknowledged that no structured literature currently supports a direct translation of Qp/Qs concepts to the context of oxygenators and pumps. Nevertheless, raising this comparison may stimulate further studies on the relationship between extracorporeal propulsion technology and internal flow heterogeneity. In conclusion, the design of hollow-fiber oxygenators, whether cylindrical, parallelepiped, spiral, radial, or cross-flow, inevitably shapes the extent and clinical impact of shunt and recirculation. Yet equally important is the way we monitor and interpret these hidden flows, using flowmeters and pressure transducers in the setting of roller or centrifugal pumps [2, 3]. Recognizing and quantifying shunt phenomena could enrich our understanding of oxygenator behavior, much as Qp/Qs analysis transformed our approach to congenital cardiac shunts. Opening this perspective may lead to new insights into the interaction between device design, pump technology, and perfusion practice, ultimately improving safety and efficiency in extracorporeal support.

## References

[1] Liu Y, Zhu Y, Yu J, Wang S, Yang M. Effect of pulsatile blood flow parameters on membrane oxygenator performance: a cross-scale simulation study. Int J Numer Meth Biomed Eng. 2025;41(7):e70076. 10.1002/cnm.70076. 40695462

[2] Segers PA, Heida JF, de Vries I, Maas C, Boogaart AJ, Eilander S. Clinical evaluation of nine hollow-fibre membrane oxygenators. Perfusion. 2001;16(2):95–106. 10.1177/026765910101600203. 11334201

[3] Visser C, de Jong DS. Clinical evaluation of six hollow-fibre membrane oxygenators. Perfusion. 1997;12(6):357–368. 10.1177/026765919701200604. 9413848

